# Improvement of Heating Uniformity by Limiting the Absorption of Hot Areas in Microwave Processing of CFRP Composites

**DOI:** 10.3390/ma14247769

**Published:** 2021-12-16

**Authors:** Shengping Li, Yingguang Li, Jing Zhou, Youyi Wen

**Affiliations:** 1 National Key Laboratory of Science and Technology on Helicopter Transmission, Nanjing University of Aeronautics and Astronautics, Nanjing 210016, China; ShengpingLi@nuaa.edu.cn (S.L.); zhoujing@nuaa.edu.cn (J.Z.); 2 AVIC Chengdu Aircraft Industrial (Group) Co., Ltd., Chengdu 610092, China; Youyi_wen@163.com

**Keywords:** microwave processing, heating uniformity, carbon fiber, polymer matrix composites

## Abstract

Carbon fiber reinforced polymer (CFRP) composites are integral to today’s industries. Curing or consolidation are vital processes for manufacturing CFRP components. Microwave processing has many advantages compared with conventional processing technologies using ovens or autoclaves; however, the uneven temperature distribution caused by the non-uniform microwave field has a significant influence on the quality of the cured products. In this study, we propose a new idea to solve this problem, i.e., limiting the absorption of hot areas. Under such circumstances, cold ones can catch up with them more easily. To adjust the absorbing capability of the CFRP laminate, periodically arranged metallic resonance structures supported by a dielectric spacer are introduced on its surface. The dielectric spacer, made of epoxy matrix and strontium titanate particles, is designed to possess a dielectric constant positively related to temperatures. In this situation, the microwave absorption (2.45 GHz) of the metal-dielectric-CFRP configuration is changed from 97.6% at room temperature to 55.9% at 150 °C continuously. As a result, a reduction of 43.1% in maximum temperature difference and 89% in standard deviation has been achieved.

## 1. Introduction

Carbon fiber reinforced polymer (CFRP) composites are integral to today’s aerospace, automotive, and energy industries due to their excellent specific strength and stiffness, chemical resistance, and thermal stability [1]. Curing or consolidation are vital processes when manufacturing CFRP components [2,3], where the resin matrix is changed from the viscous state in preforms to the loadable solid phase in final products, through chemical reactions or physical melting and solidification at specific temperatures (usually between 100~300 °C). Currently, CFRP components are commonly heated by heat transfer, in ovens and autoclaves, or via tools (in liquid composite molding), which suffers from issues such as inaccurate temperature control, long processing cycle, and sometimes high energy consumption [4,5]. Inversely, microwave heating has many advantages in these aspects [6], and it is considered a promising alternative for composites processing [7,8,9]. However, the uneven temperature distribution seriously restricts the industrial application of the microwave processing technology, as problems such as distortion and ablation would be caused by the local hot spots and cold spots on the composite surface [10,11,12]; therefore, how to improve the heating uniformity is of great significance for composites microwave processing and has attracted much attention worldwide.

Existing methods to improve the microwave heating uniformity can be divided into three categories. The first one focuses on the design of the resonant cavity and microwave sources beforehand, e.g., changing the cavity shape, increasing the number of microwave sources, etc. [13,14]. The purpose is mainly to obtain more electromagnetic modes, as they may have complementary effects [13]. The second one is to generate a relative movement between the electromagnetic field and the object being heated via rotating trays, reciprocating mode agitators, and variable frequency microwave sources [15,16,17]. For example, rotating trays are widely used in household microwave ovens, on which the food may move through areas of high and low power fields alternately. Thus, time-averaged-uniformity may be realized, but, of course, the situation may become even worse sometimes, based on the random compensation principle. Unlike the above methods, the third one dedicates to establishing an active compensation process based on real-time temperature monitoring. For example, Zhou et al. reported a multi-pattern compensation method in which the relationship between the composite heating pattern and the power ratio of multiple microwave sources were described by a database to compensate for the uneven temperature monitored by adjusting the outputs of the microwave sources accordingly [18]. Later, they further proposed an online learning-based temperature control method that established the relationship between the heating pattern and the power ratio of microwave sources in real-time by machine learning instead of constructing a database beforehand [19]. Although the active compensation methods have improved the microwave heating uniformity effectively, they rely on the development of temperature measurement methods, especially those compatible with the complex processing environment of actual products.

In this paper, for the first time, we report a new idea to improve the microwave heating uniformity of CFRP laminates by limiting the absorption of hot areas. Under such circumstances, cold ones can catch up with them more easily. To demonstrate this, periodically arranged metallic resonance structures supported by a dielectric-variable spacer are introduced on the CFRP laminate. For convenience, the resonance structure supported by a dielectric-variable spacer is called ReSDiV film. A series of simulations and experiments have been performed to design the ReSDiV film. On this basis, microwave absorption (2.45 GHz) negatively related to temperatures has been demonstrated at both normal and oblique incidence. The effectiveness in improving heating uniformity has been validated.

## 2. Overview of Our Method

**Figure 1:**
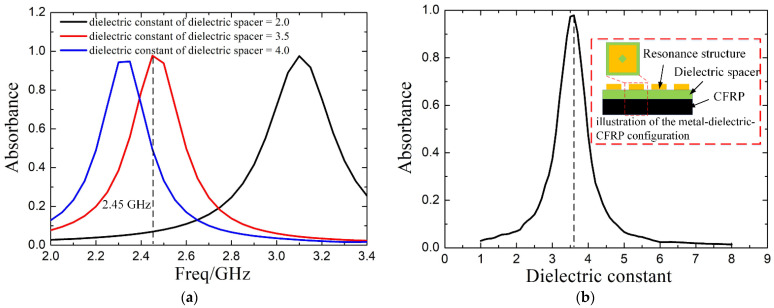
(**a**) The frequency-dependent simulated absorbance of the metal-dielectric-CFRP configuration with different dielectric constants of the dielectric spacer (2, 3.5, and 4); (**b**) the simulated absorbance of the metal-dielectric-CFRP configuration with the change of the dielectric constant of the dielectric spacer at 2.45 GHz.

**Figure 2:**
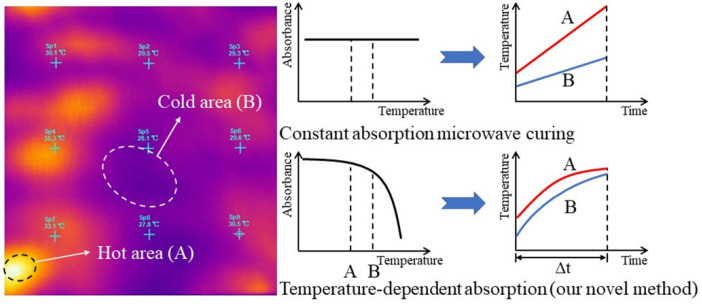
Schematic diagram of our novel method.

In our previous works, we made the metal-like CFRP laminates remarkably absorptive by employing metallic resonance structures supported by a dielectric spacer [20,21]. Through further explorations, we find that the dielectric constant of the dielectric spacer has a significant impact on the microwave absorption of the metal-dielectric-CFRP configuration. As shown in Figure 1a, by adjusting the dielectric constant of the dielectric spacer from 3.5 to 2.0 and 4.0, the central frequency of the absorption peak shifts from 2.45 GHz to 2.35 GHz and 3.1 GHz, respectively. The absorbance (the fraction of incident power absorbed) of the metal-dielectric-CFRP configuration with the dielectric constant of the dielectric spacer was obtained based on the simulation model in [20]. The resonance structures (copper) are an array of subwavelength square cells with a diamond hole in the center. The dielectric spacer is a polyimide film of which the dielectric constant is about 3.5. Take 2.45 GHz as an example. By changing the dielectric constant of the dielectric layer from 1 to 8, the absorbance reaches the maximum at 3.6, then it decreases monotonously, as shown in Figure 1b. Under such circumstances, we suppose that the heating uniformity may be improved significantly if the absorbance of the metal-dielectric-CFRP configuration is adjusted to decrease monotonously with temperature. This is because the heating rate of hot areas on the laminate will be limited. Thus, cold ones can catch up with them in an easier way, thereby improving the temperature uniformity of the laminate (see Figure 2).

From the literature survey, we found that strontium titanate (STO) has temperature-dependent dielectric properties, but its dielectric constant is too high (>150 at 2.45 GHz between 25 °C and 200 °C [22,23]). As a result, STO particles were added into a low dielectric epoxy resin matrix to fabricate our dielectric-variable spacer. After that, the waveguide method was adopted to evaluate the temperature-dependent dielectric properties of the spacer. The resonance structures were designed and prepared accordingly. Related details will be introduced in subsequent sections.

## 3. Materials and Methods

### 3.1. Materials

A unidirectional prepreg (Weihai Guangwei composite material Co., Ltd., Weihai, China), made of epoxy resin and polyacrylonitrile-based T800 carbon fibers, was used to fabricate the CFRP laminate. The curing temperature of the matrix resin is 150 °C, and the thickness is 0.1 mm. Adhesive-less flexible copper-clad polyimide film was used to fabricate the resonance structures. SrTiO_3_ (STO, the CAS number is 12060-59-2) ceramic particles were purchased from Quanzhou Qijing New Material Technology Co., Quanzhou, China. According to the supplier’s datasheet, the diameter of the STO particles was 1 μm. A two-component epoxy (NM503, Kunshan Lvxun Chemicals Co., Kunshan, China) was chosen for its temperature resistance of 200 °C. Other auxiliary materials, such as peel ply, release film, ventilate felt, and vacuum bag, were purchased from Airtech.

### 3.2. Sample Preparation

**Figure 3:**
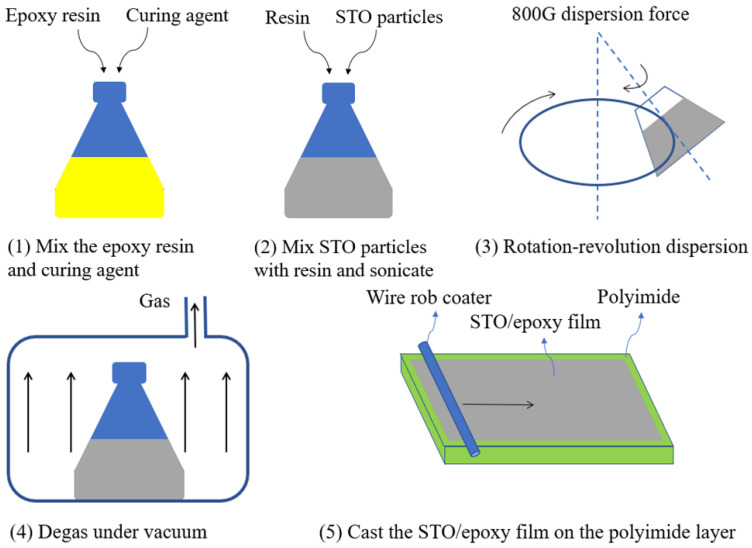
Flow chart of STO/epoxy film fabrication.

Firstly, the copper foil on the copper-clad polyimide film was cut to a specific shape using a laser-printed circuit board etcher (LPKF ProtoLaser U4), forming the resonance structures. Then STO particles were added to the epoxy resin forming the STO/epoxy film. Figure 3 presents the flow chart of the STO/epoxy film fabrication process. Since it is difficult to guarantee uniformity and formability when the mass fraction of STO particles exceeds 50%, STO/epoxy films with STO mass fractions of 10%, 20%, 30%, 40%, and 50% were prepared in this work [24]. After that, the unidirectional prepreg was cut into square pieces of a specific size to fabricate orthogonal laminates ([0°/90°]_10_) with the ply angle of 0° and 90°. Finally, the resonance structure, dielectric-variable spacer, and CFRP laminate were stacked and attached by vacuum, forming the samples for microwave absorption measurement or microwave heating experiment.

### 3.3. Dielectric and Absorbing Property Measurement

The temperature-dependent dielectric properties of the STO/epoxy film were measured by the waveguide method [25]. Specifically, the STO/epoxy film was heated by conduction to a specific temperature which varied from room temperature (25 °C) to 150 °C with an interval of 10 °C. Then, it was quickly put into a BJ26 waveguide, and the corresponding dielectric properties were measured immediately by a vector network analyzer (Keysight N5244B). The temperature-dependent absorbance of the metal-dielectric-CFRP configuration was tested through the free space method [26]. The size of the CFRP laminate used was 200 × 200 × 2 mm^3^. The ReSDiV film-covered CFRP laminate was placed in front of a horn antenna. An electro-thermal film was attached behind the CFRP laminate for in situ heating, as no transmission existed at that position. In this configuration, the reflectivity (the fraction of incident power reflected) at various temperatures was measured, and the absorbance was calculated accordingly.

### 3.4. Microwave Heating Experiment

**Figure 4:**
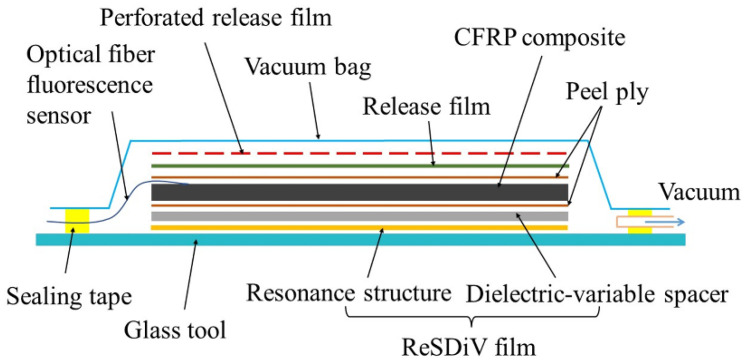
The vacuum-packed configuration of samples for the heating experiment.

Figure 4 shows the vacuum packaging configuration of samples for microwave heating experiments. The size of the [0°/90°]_10_ CFRP laminate was 250 × 250 × 2 mm^3^. As shown, the resonance structures, dielectric-variable spacer, release film, CFRP laminate, release film, perforated release film, breather, and vacuum bag were placed on a glass tool in sequence and sealed by a high temperature-resistant sealing tape. Further, nine optical fiber fluorescence sensors (Beijing Oriental Ruize Technology Co., Ltd., Beijing, China) were distributed evenly on the upper surface of the CFRP laminate for temperature monitoring. Each of them consists of an optical fiber, a fluorescent light source, and a protective cover with a diameter of 2 mm. Wave-transparent ventilate felts were used for heat preservation. An octagonal microwave oven, developed by our team, was used to heat the CFRP laminates [27]. During the heating process, the difference between the average temperature and the setting value was used to regulate the output power of the microwave source through the PID algorithm. For comparison, two experiments were conducted. The first one used the ReSDiV film developed in this paper, i.e., resonance structures supported by the dielectric-variable spacer. In the other experiment, the resonance structures were supported by a dielectric pacer with constant dielectric properties [20]. For each configuration, the experiment was repeated three times. In addition, we conducted another set of experiments without the ventilate felt for heat preservation to capture the thermal images during the heating process through a FLIR A300 thermal imager.

## 4. Results and Discussion

### 4.1. Design of the Dielectric-Variable Spacer

**Figure 5:**
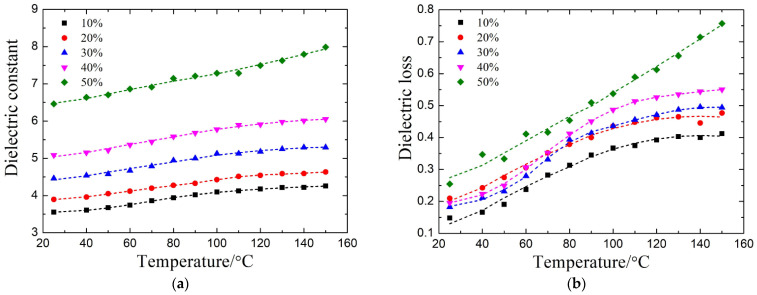
(**a**) The dielectric constant and (**b**) dielectric loss of the STO/epoxy films with the 10%, 20%, 30%, 40%, and 50% STO mass fraction at 2.45 GHz.

Figure 5a shows the dielectric constant of the STO/epoxy film at 2.45 GHz. As shown, the dielectric constant increases with the STO mass fraction. For example, the dielectric constants of films with STO mass fractions of 10%, 20%, 30%, 40%, and 50% at room temperature are 3.552, 3.895, 4.463, 5.084, and 6.461, respectively. This is because the dielectric constant of the STO (>150 at 2.45 GHz [22,23]) is much higher than that of the resin (about 3.5 at 2.45 GHz [28]). Further, for an STO/epoxy film with a specific STO mass fraction, the dielectric constant shows a monotonical increase from room temperature to 150 °C. It may result from resin mobility increase caused by temperature rise [29]. The dielectric constant of the STO/epoxy film with a 50% mass fraction of STO increases from 6.461 to 7.987 at the temperature range, exhibiting the highest increase as a function of temperature. Thus, it can realize the maximum variation in the microwave absorption of the metal-dielectric-CFRP configuration when selected as the dielectric-variable spacer. Figure 5b depicts the dielectric loss of the STO/epoxy film at 2.45 GHz, which exhibits similar behavior to the dielectric constant. Even so, the dielectric loss is relatively low. As a result, the high dissipative CFRP substrate rather than the dielectric-variable spacer can absorb the most microwave energy [30]. As mentioned above, the STO/epoxy film with 50% STO mass fraction is selected as the dielectric-variable spacer to construct the ReSDiV film.

### 4.2. Design of the ReSDiV Film

**Figure 6:**
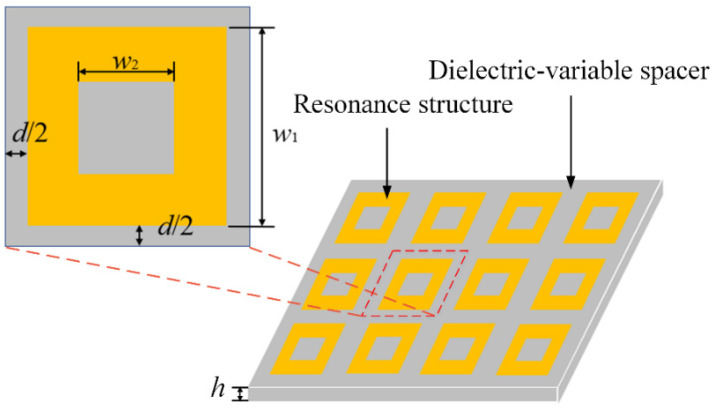
The schematic diagram of the ReSDiV film.

Figure 6 shows the schematic of the adopted ReSDiV film. The resonance structures are periodically arranged copper square rings, and the dielectric-variable spacer is the STO/epoxy (wt.50%) film. To obtain a remarkable variation in the absorbance within the heating temperature (25~150 °C), the ReSDiV film should be designed to make the metal-dielectric-CFRP configuration possess a strong absorption at room temperature. Under this condition, frequency-dependent simulations were performed to optimize the dimensions of the ReSDiV film (*w*_1_, *w*_2_, *d*, and *h* in Figure 6). Specifically, a single-port model was constructed in the High-Frequency Structure Simulator (HFSS) [20]. A plane wave was incident on an infinite metal-dielectric-CFRP configuration using periodic boundaries. A perfect electric conductor boundary was defined on the bottom of the metal-dielectric-CFRP configuration. In this way, the absorbance can be calculated using the ratio of the reflected signal to the incident signal. That is, the less power reflected, the more is absorbed, and vice versa, as there is a zero transmittance in our case. As mentioned above, the dimensions of the ReSDiV film were optimized: $w_{1}=24.25\;\mathrm{mm}$, $w_{2}=11\;\mathrm{mm}$, d=1 mm, and h=0.52 mm.

**Figure 7:**
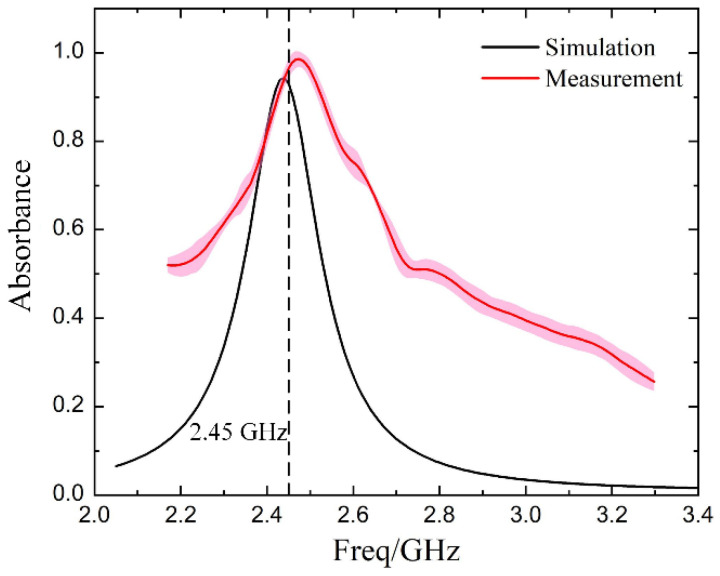
The frequency-dependent (simulated and measured) absorbance of the metal-dielectric-CFRP configuration. The absorbance at 2.45 GHz is at the intersection of the curve and the black dashed line. The pink area indicates the error band of the measured results.

Figure 7 shows the frequency-dependent absorbance of the CFRP laminate, covered by the fabricated ReSDiV film, at room temperature. Among them, the measured results were obtained from three repeated measurements to reach an average. It can be observed that the absorption peaks of the simulation and the experiment are both around 2.45 GHz, and the values at 2.45 GHz are also very close (93% in simulation, 97.6% in measurement). This verifies the effectiveness of the designed ReSDiV film very well. The slight difference between the simulation and test may come from two aspects. On the one hand, the test error of the dielectric properties of the STO/epoxy film is mainly responsible for the deviation between the simulation and test. On the other hand, the fabrication tolerances and the test precision of the microwave absorption may also have some influence.

### 4.3. Validation of Temperature-Dependent Microwave Absorption

**Figure 8:**
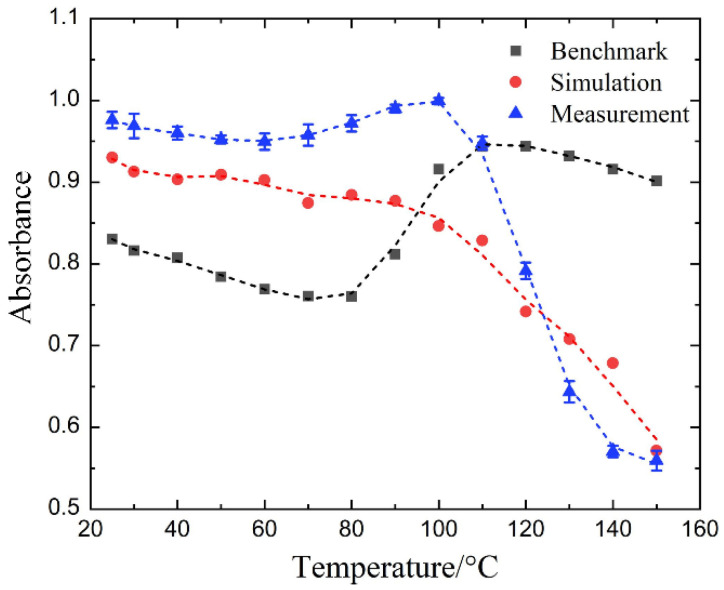
The temperature-dependent (simulated and measured) absorbance of the metal-dielectric-CFRP configuration at normal incidence at 2.45 GHz. The error bars in the blue curve indicate the standard deviation of the three repeated measurements. The benchmark shows the measured result of the CFRP laminate capped with the resonance structures supported by a dielectric spacer with constant dielectric properties (black line).

**Figure 9:**
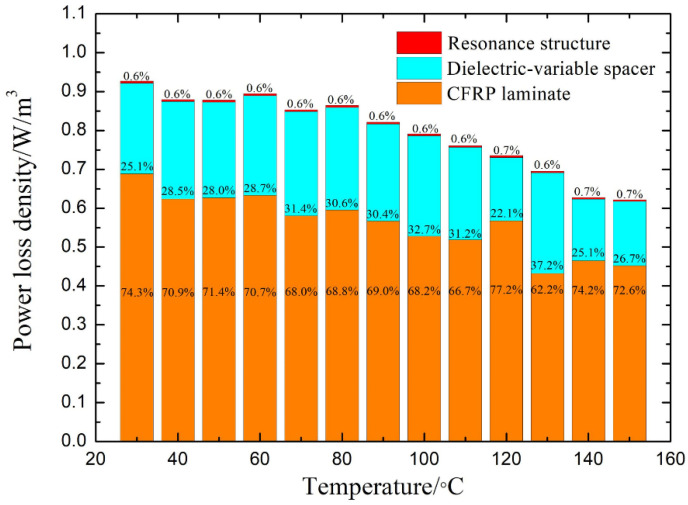
The power loss density of the metal-dielectric-CFRP configuration at normal incidence at 2.45 GHz. The percentage value represents the proportion of the energy loss of this part in that of the entire configuration.

Simulations and measurements have been conducted to investigate the temperature-dependent microwave absorbing property (2.45 GHz) of the metal-dielectric-CFRP configuration. In the test, each temperature point was measured three times to obtain the average value as the final result. Figure 8 presents the corresponding results at normal incidence. As shown, the measured results exhibit a similar trend to the simulated results. The measured absorbance decreases from 97.6% at 25 °C to 55.9% at 150 °C, and the simulated one decreases from 93% to 57.1%. Particularly, both of them decrease drastically from 100 °C to 150 °C. In addition, the measured result of the CFRP laminate capped with the resonance structures supported by a dielectric spacer with constant dielectric properties is also shown in the figure as a benchmark, which fluctuates slightly as the temperature rises yet remains at a high level (>75%). Thus, the reduction in microwave absorption has been realized when the temperature varies from room temperature to 150 °C. In the meantime, the field calculator in HFSS was used to obtain the power loss density inside the metal-insulator-CFRP configuration. As shown in Figure 9, the energy loss of the entire configuration shows the same decrease with the absorbance as the temperature rises from 25 °C to 150 °C. The CFRP laminate is the one inside the metal-insulator-CFRP configuration that absorbs the most microwave energy. More than 62.19% of the absorbed energy is dissipated in the CFRP laminate. The energy loss of the metallic resonance structure (less than 7.1%) is much smaller than that of the CFRP laminate. By comparison, the dielectric loss of the dielectric-variable spacer (22.1~37.2%) is more considerable, which can be further suppressed by utilizing new lossless materials in the future.

**Figure 10:**
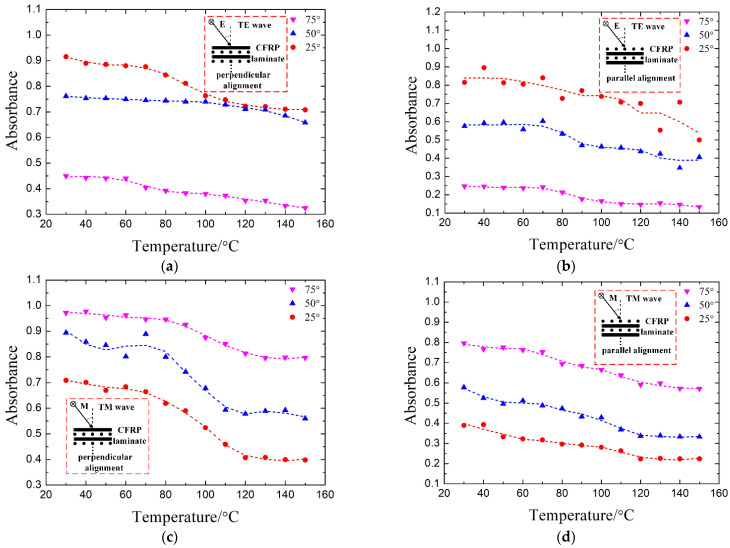
The temperature-dependent simulated absorbance of the metal-dielectric-CFRP configuration at oblique incidence (25°, 50°, and 75°) at 2.45 GHz. The absorbance of TE (**a**,**b**) and TM (**c**,**d**) wave in the case of perpendicular alignment (**a**,**c**) and parallel alignment (**b**,**d**).

In a microwave cavity, the electromagnetic waves are embodied as the Transverse Electric (TE) and Transverse Magnetic (TM) waves. They are incident on the surface of the material from different angles, and the electric and magnetic components distribute in various directions, which can be decomposed into perpendicular to (perpendicular alignment) and parallel to (parallel alignment) the fiber axis of the first lamina of the CFRP laminate ([0°/90°]_10_). Figure 10 exhibits the absorbance of the metal-dielectric-CFRP configuration under various incidence angles of both TE and TM waves, in the case of parallel alignment and perpendicular alignment. In general, in all cases, the absorbance shows a downward trend with increasing temperature. For example, in the case of a 25° incident TM wave and perpendicular alignment, the absorbance decreases by 21.5% with temperature. In addition, in the case of the TE wave, the magnitude of the reduction in absorbance decreases with the increase in angle. This may be because the magnetic field component paralleling the CFRP laminate decreases as the angle increases, which has a huge impact on the absorbance [20,31]. As mentioned above, our metal-dielectric-CFRP configuration can work very well in the microwave cavity.

### 4.4. Microwave Heating Performance

**Figure 11:**
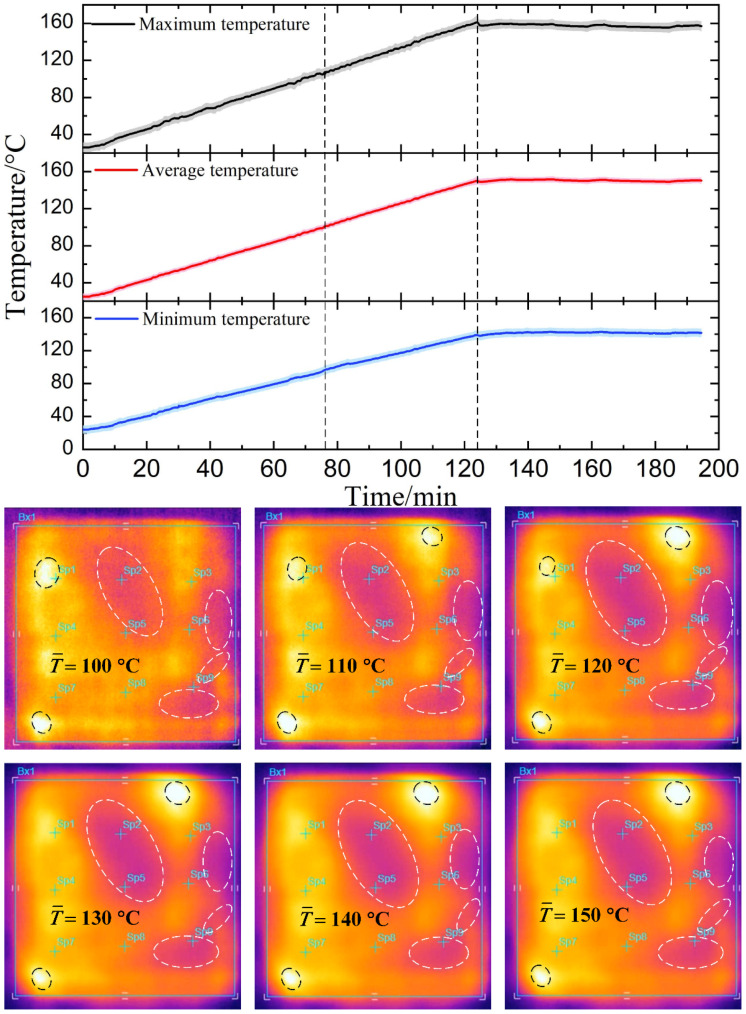
The statistical temperature profiles in the case of constant microwave absorption. The infrared thermal images show the temperature distribution during the heating-up period from 100 °C to 150 °C (The area between the two black dashed lines; the hottest and coolest spots are marked by the black and white dashed box, respectively).

To demonstrate the effectiveness of our method, microwave heating experiments were conducted on CFRP laminates capped with the resonance structures supported by a dielectric spacer with constant and temperature-dependent dielectric properties. For convenience, the two kinds of samples are termed laminate with constant and temperature-dependent absorption, respectively. The ramp rate of the heating process was set as 1 °C/min, and the target temperature was 150 °C. After that, the temperature was maintained for 1 h. During this process, the average temperature acted as the object controlled. The statistical temperature profiles in the case of constant microwave absorption are shown in Figure 11. In the heating stage, the slope of the maximum temperature curve is always larger than the minimum temperature, resulting in a gradual increase in the maximum temperature difference. As dwell progressed, the maximum temperature difference descends to some extent because of heat conduction inside the material. It can be observed that the positions of the hot and cold spots were almost unchanged during the whole process. These thermal images, from 100 °C to 150 °C, were captured by a FLIR imager on another sample without heat preservation. The unchanged hot and cold spots led to the continuous deterioration of temperature uniformity.

**Figure 12:**
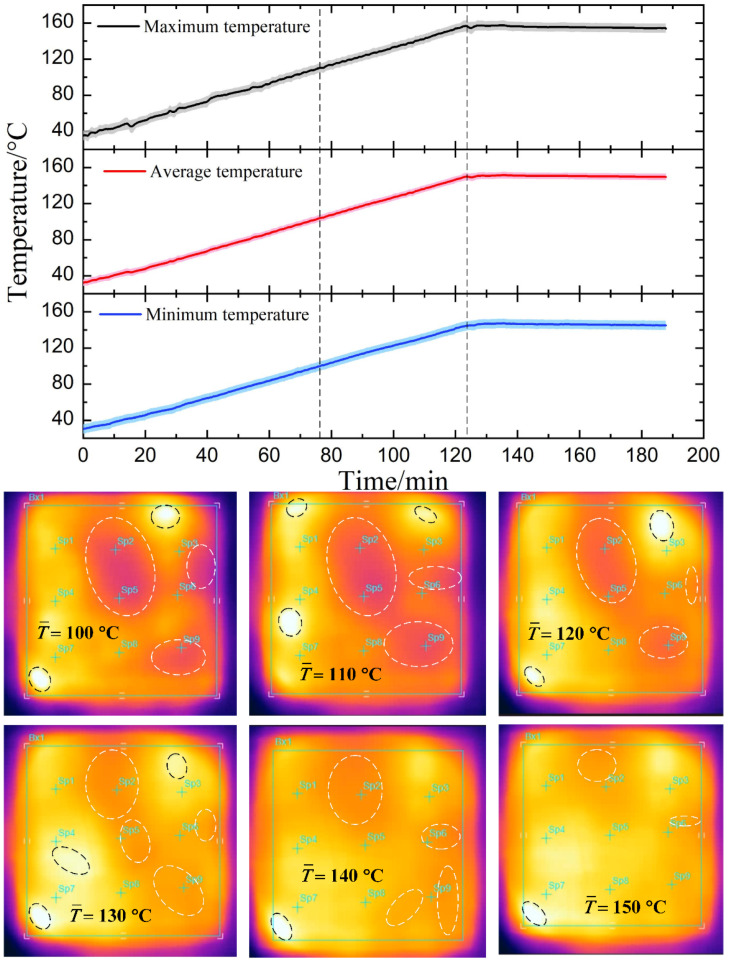
The statistical temperature profiles in the case of temperature-independent microwave absorption. The infrared thermal images show the temperature distribution during the heating-up period from 100 °C to 150 °C (The area between the two black dashed lines; the hottest and coolest spots are marked by the black and white dashed box, respectively).

Figure 12 presents the statistical temperature profiles in the case of temperature-dependent microwave absorption. In the heating-up period, the maximum (minimum) temperature of the laminate is closer to the average temperature than that of the laminate with constant absorption. The slope of the maximum temperature curve is slightly larger than the minimum temperature, and the two curves are almost parallel in certain temperature sections. The infrared thermal images from 100 °C to 150 °C show that the positions of the hot and cold spots continue to change, and the in-plane temperature tends to be uniform as the temperature rises. Because the temperature-dependent absorption of the metal-dielectric-CFRP configuration can limit the heating speed of the hot spots, so that the temperature of the cold spots approaches the hot spots gradually, improving the heating uniformity.

**Figure 13:**
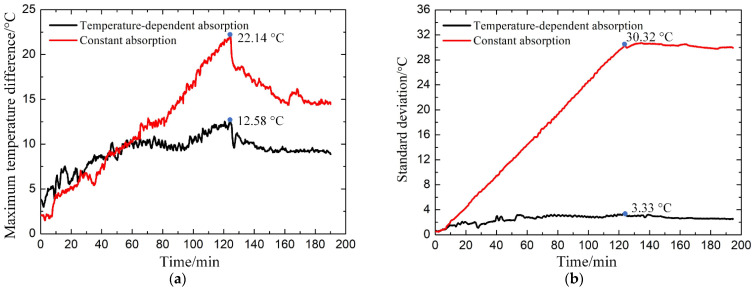
Statistics of the (**a**) maximum temperature difference and (**b**) standard deviation of the laminates with variable absorption with temperature (black line) and constant (red line) absorption.

The maximum temperature difference for the two heating processes is summarized in Figure 13a. For the laminate with constant absorption, the maximum temperature difference increases almost linearly in the heating stage and then decreases to stable in the dwelling stage. The initial drop in the dwelling stage is due to the heat transfer from hot spots to cold spots in the laminate. Later a balance is achieved between the heat generated by the laminate and the heat loss to the external environment, making the maximum temperature difference stable. For the laminate with temperature-dependent absorption, the maximum temperature difference increases much slower due to the temperature-dependent absorption, especially in the temperature section from 100 to 150 °C. At the beginning of the dwelling stage, the maximum temperature difference reaches the maximum value of 12.58 °C, compared to 22.14 °C with constant absorption, which shows a reduction of 43.1%. As a result, the temperature distribution of the laminate with temperature-dependent absorption is more homogeneous than that with constant absorption. In addition, it can be demonstrated by the standard deviation in the two heating processes shown in Figure 13b. The standard deviation of the laminate with temperature-dependent absorption gradually stabilizes at 3.33 °C at the end of the heating-up period, while the other reaches 30.32 °C; thus, a reduction of 89% has been achieved. As mentioned above, the effectiveness of the presented method has been demonstrated. It can be further optimized by considering the processing parameters of the composite in the future.

## 5. Conclusions

In summary, we propose an idea to improve the temperature uniformity of CFRP composites during microwave curing by limiting the absorption of hot areas so that cold areas can catch up with them more easily. It was realized by introducing periodically arranged metallic resonance structures supported by a dielectric-variable spacer on the CFRP laminate. Decreasing absorption with increasing temperature has been achieved at normal incidence, and it is robust against incidence angles for TE and TM waves. To ensure the feasibility of this method, the microwave heating experiment has been performed. Compared with the metal-dielectric-CFRP configuration with constant absorption, the maximum temperature difference and standard deviation using our method are reduced by 43.1% and 89%, respectively. The effectiveness can be further optimized by considering the processing parameters of the composite in the future.

## Data Availability

The data presented in this study are available on request from the corresponding author.
